# Chromatin accessibility and translational landscapes of tea plants under chilling stress

**DOI:** 10.1038/s41438-021-00529-8

**Published:** 2021-05-01

**Authors:** Pengjie Wang, Shan Jin, Xuejin Chen, Liangyu Wu, Yucheng Zheng, Chuan Yue, Yongchun Guo, Xingtan Zhang, Jiangfan Yang, Naixing Ye

**Affiliations:** 1 College of Horticulture, Fujian Agriculture and Forestry University/Key Laboratory of Tea Science in Universities of Fujian Province, Fuzhou, 350002 China; 2 Shenzhen Branch, Guangdong Laboratory for Lingnan Modern Agriculture, Genome Analysis Laboratory of the Ministry of Agriculture, Agricultural Genomics Institute at Shenzhen, Chinese Academy of Agricultural Sciences, Shenzhen, 518120 China

**Keywords:** Abiotic, Gene regulation, RNA sequencing

## Abstract

Plants have evolved regulatory mechanisms at multiple levels to regulate gene expression in order to improve their cold adaptability. However, limited information is available regarding the stress response at the chromatin and translational levels. Here, we characterize the chromatin accessibility, transcriptional, and translational landscapes of tea plants in vivo under chilling stress for the first time. Chilling stress significantly affected both the transcription and translation levels as well as the translation efficiency of tea plants. A total of 3010 genes that underwent rapid and independent translation under chilling stress were observed, and they were significantly enriched in the photosynthesis-antenna protein and phenylpropanoid biosynthesis pathways. A set of genes that were significantly responsive to cold at the transcription and translation levels, including four (+)-neomenthol dehydrogenases (MNDs) and two (*E*)-nerolidol synthases (NESs) arranged in tandem on the chromosomes, were also found. We detected potential upstream open reading frames (uORFs) on 3082 genes and found that tea plants may inhibit the overall expression of genes by enhancing the translation of uORFs under chilling stress. In addition, we identified distal transposase hypersensitive sites (THSs) and proximal THSs and constructed a transcriptional regulatory network for tea plants under chilling stress. We also identified 13 high-confidence transcription factors (TFs) that may play a crucial role in cold regulation. These results provide valuable information regarding the potential transcriptional regulatory network in plants and help to clarify how plants exhibit flexible responses to chilling stress.

## Introduction

Plants worldwide are often challenged by various environmental conditions that threaten their growth, distribution, and yield, and low temperature is one of their major limitations. With the intense temperature cycles between warm and cold seasons, plants from temperate regions have evolved multilevel regulatory mechanisms to regulate gene expression to improve their cold adaptation^1^. Current evidence shows that most changes in plants under cold stress are due to the expression of many *cold-responsive* (*COR*) genes^2–4^, and these *COR* genes are modulated by a variety of regulatory factors, such as C-repeat binding factors (CBFs)^5–7^. However, although the regulatory mechanism of the cold response in plants has been extensively studied at the mRNA level, regulation at the chromatin and posttranscriptional levels remain to be explored.

Accessible chromatin regions (ACRs) reflect the ability of chromatin to regulate gene expression, and transcription factors (TFs) can affect the occupancy of nucleosomes through dynamic interactions with *cis*-regulatory elements (CREs)^8–10^. To detect chromatin accessibility and TF binding, DNase I sensitivity coupled with high-throughput sequencing (DNase-seq) has been used in plants to identify various genome-wide *cis*-regulatory DNA elements and construct regulatory networks^11–16^. Although this method is powerful, it has recently been mostly replaced by the assay for transposase-accessible chromatin with sequencing (ATAC-seq)^17^. This method employs high-activity Tn5 transposase to simultaneously cut nuclear DNA and insert the sequencing adapter and has the advantages of low input material requirements, high sensitivity, and greatly simplified operation steps^18,19^. Notably, recent studies have focused on distal ACRs (dACRs) in plants through ATAC-seq and some chromatin assays and found that long-range transcriptional regulation by CREs is common in angiosperms^20,21^. However, to date, chromatin accessibility profiling using ATAC-seq has been mainly performed on model plants, such as *Arabidopsis*^22–24^ and rice^25–27^, and has never been applied to woody plants.

Translational control also plays a vital role in the regulation of gene expression. Increasing evidence suggests that large discrepancies between the levels of mRNA and the corresponding proteins may exist in plants^28–30^, which highlights the importance of studying the translational regulation of plant gene expression. Ribosome profiling sequencing (Ribo-seq) is a recently developed high-throughput technology that can accurately monitor the translation process by sequencing the ribosome footprints (RFs) mapped on mRNA^31,32^. With the rapid application of Ribo-seq technology in plants, translational regulation has been revealed to play an important role in plant responses to several biotic and abiotic stimuli, including pathogens, hypoxia, light, ethylene, drought, and heat stress^28,29,33–39^. However, whether translational reprogramming is widely involved in the plant response to low-temperature stress and how it affects the translation efficiency of transcripts in plants remain unknown.

The tea plant, *Camellia sinensis* L. O. Kuntze, is an important perennial woody plant that is widely cultivated in subtropical to tropical climate regions. Cold stress is one of the most serious environmental challenges faced by tea plants^40^. Previous studies have elucidated the cold resistance mechanism of tea plants at the mRNA level^41–44^, characterized the functions of volatiles and related genes at low temperatures^45,46^, and found that CBFs may play a functional role by regulating target genes in several pathways under natural winter cold stress^47^. In this study, we initially determined the chromatin accessibility, transcriptional, and translational landscapes of tea plants in vivo under chilling stress by combining ATAC-seq, RNA-seq, and Ribo-seq technologies. These results will provide new insights into plant gene regulation under chilling stress.

## Results

### Overview of ATAC-seq, RNA-seq, and Ribo-seq

**Fig. 1:**
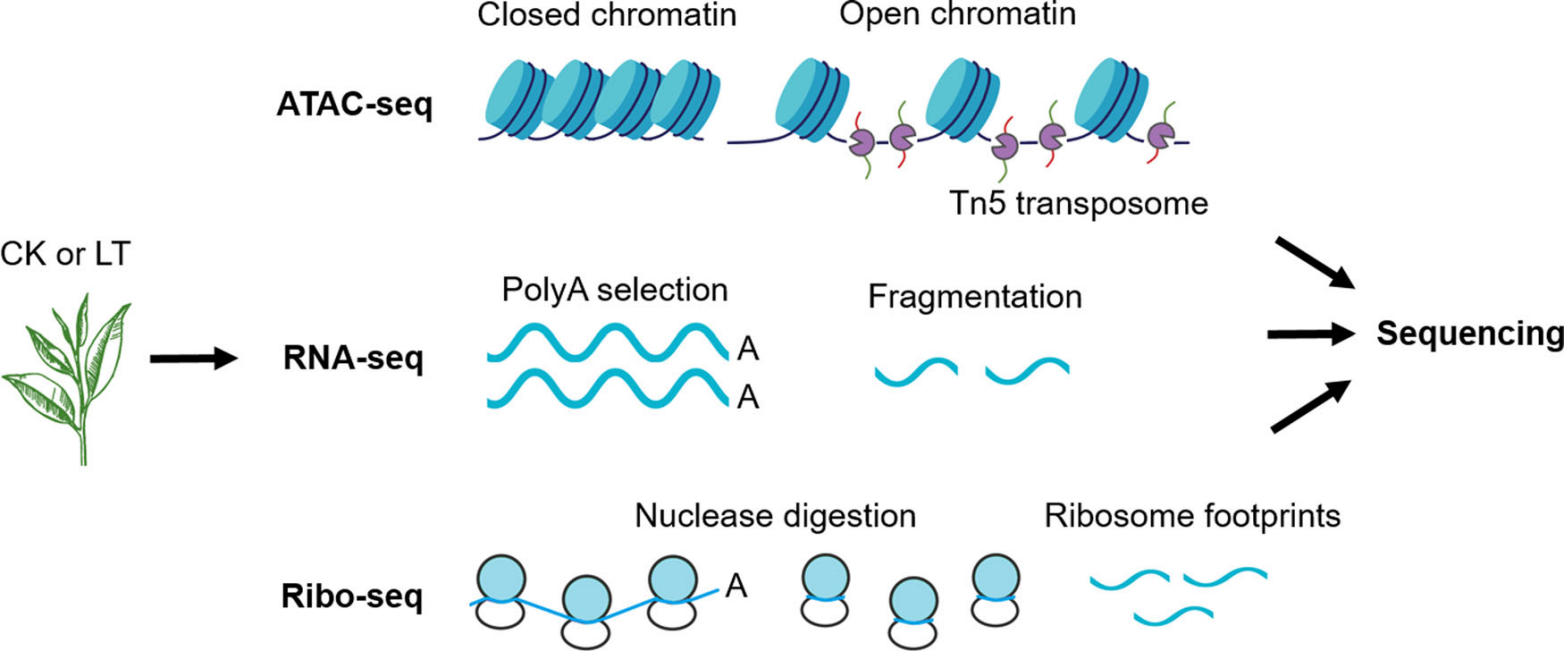
Schematic representation of the experimental strategy. The chromatin accessibility, transcriptional, and translational landscapes of tea plants in vivo under chilling stress were determined by combining ATAC-seq, RNA-seq, and Ribo-seq technologies

To investigate the regulation of chilling stress responses in tea plant leaves from chromatin accessibility, transcriptional, and translational aspects, we collected samples from the control (CK) and the plants treated at 4 °C for 24 h (low temperature (LT)) and performed whole-genome ATAC-seq, RNA-seq and Ribo-seq analysis in vivo (Fig. 1). A total of 69.23–87.30 million ATAC-seq clean reads (Table S1), 37.70–43.20 million RNA-seq clean reads (Table S2), and 132.13–163.20 million Ribo-seq clean reads (Table S3) were obtained for the CK and LT samples, respectively. The Q20 and Q30 of the clean reads of the three omics methods exceeded 96% and 91%, respectively, and 88.05–97.87% of clean reads were mapped to the latest tea plant genome (cv. Huangdan, https://bigd.big.ac.cn/search/?dbId=&q=CRA003208). The qRT-PCR results were well correlated with the RNA-seq data (Fig. S1). Principal component analysis (PCA) clearly distinguished the CK and LT samples by chromatin, transcription, and ribosome profiling (Figs. S2–S4). These results all show that the three omics approaches we applied are highly reliable and reproducible and that chilling stress modulates the transcriptional and posttranscriptional processes of tea plants to a certain extent.

### Response to cold at the transcription and translation levels

**Fig. 2:**
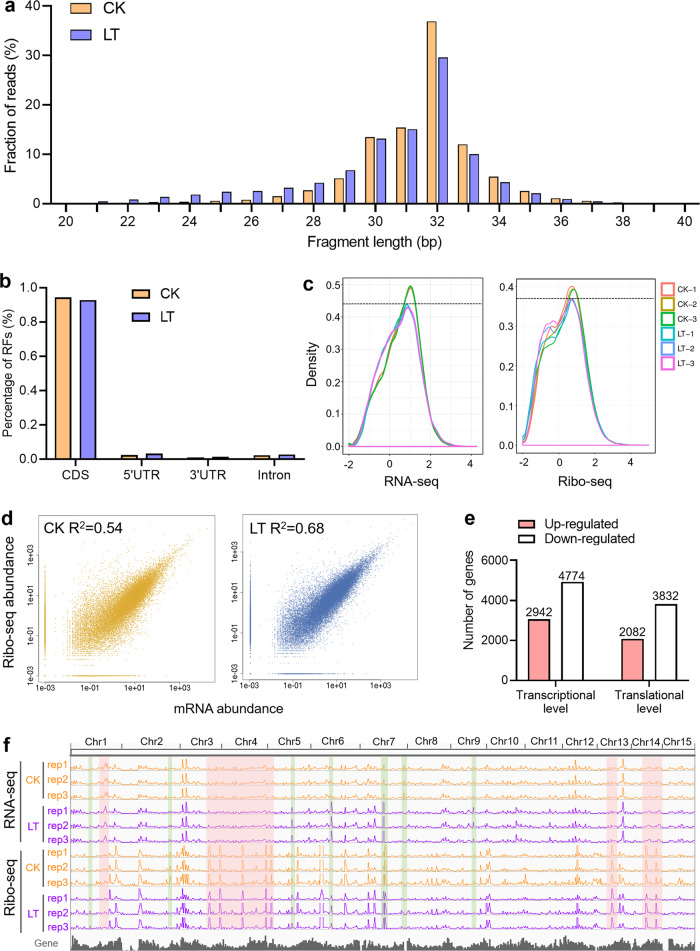
Characteristics of RNA-seq and Ribo-seq data in tea plant leaves under chilling stress. **a** Length distribution of ribosome footprints (RFs) in the CK and LT samples. The bars represent three biological replicates. **b** Genomic distribution of RFs from CK and LT. **c** Distribution of expression abundance from Ribo-seq and RNA-seq data. The abscissa represents log10 FPKM values, and the dotted line clearly distinguishes the transcription and translation abundances of the CK and LT groups. **d** Pearson correlation coefficients between the gene translational level and transcriptional level under chilling stress. **e** Number of genes differentially expressed at the transcription or translation level under chilling stress. **f** IGV browser view showing global RNA-seq and Ribo-seq tracks under chilling stress. Changes in the transcription or translation level caused by chilling stress are highlighted in light green shading. Significant changes in CK and LT at only the transcription or translation level are shaded in light red

We first checked several features of the ribosome profiles to further determine the quality of Ribo-seq. Our results showed that the length of RFs in the CK and LT samples was approximately 32 nt (Fig. 2a), which is similar to previous results in tea plant buds and leaves^30^ and slightly higher than the 30 nt reported in *Arabidopsis*^33^ and maize^28^ and the 28 nt reported in yeast^31^. Several studies have found that many small ORFs exist in the untranslational regions (UTRs) of eukaryotes and play important roles in regulating gene expression^28,48,49^. We observed that 2.34%, 1.05%, and 2.28% of RFs were located in the 5′ UTR, 3′ UTR, and introns, respectively, while 94.40% of RFs were located in CDSs in the CK samples (Fig. 2b). Compared with that in the CK samples, the percentage of RFs located in the 5′ UTR, 3′ UTR, and introns of the LT samples increased to 3.19%, 1.29%, and 2.72%, respectively, while the percentage of RFs mapped to CDSs dropped to 92.81% (Fig. 2b). These changes were consistent with those in maize under drought stress^28^, implying that translation in UTRs may mediate the plant response to stress.

For the RNA-seq and Ribo-seq data, the three biological replicates of the CK and LT groups were highly correlated (*R* > 0.96, Fig. S5). The high correlation was further reflected in the expression abundance distribution of Ribo-seq and RNA-seq data, and the findings also clearly showed that chilling stress inhibited the transcription and translation levels of tea plants (Fig. 2c). Interestingly, we found that the Pearson correlation coefficient between gene transcription and translation levels under cold conditions increased from 0.54 to 0.68 (Fig. 2d), which reflects the synergistic effect of transcription and translation under stress conditions. Correspondingly, the moderate correlation indicates that tea plants must have independent translation regulation under chilling stress.

Based on the criteria of an absolute fold change ≥ 2 and FDR < 0.05, we obtained 2942 upregulated and 4774 downregulated DEGs at the transcriptional level and 2082 upregulated and 3506 downregulated DEGs at the translational level (Fig. 2e). The number of upregulated genes was smaller than that of downregulated genes at both levels, indicating that chilling stress inhibits the expression of tea plant genes. We observed that DEGs at the transcription and translation levels share many enriched pathways related to chilling stress responses, such as photosynthesis (ko00195), oxidative phosphorylation (ko00190), MAPK signaling (ko04016), and plant hormone signal transduction (ko04075) pathways (Fig. S6). It is noteworthy that the global RNA-seq and Ribo-seq tracks of 15 chromosomes obviously show that chilling stress causes changes at many levels, including only at the transcriptional level, only at the translational level, and at both levels (Fig. 2f).

### Functional comparison at the transcription and translation levels

**Fig. 3:**
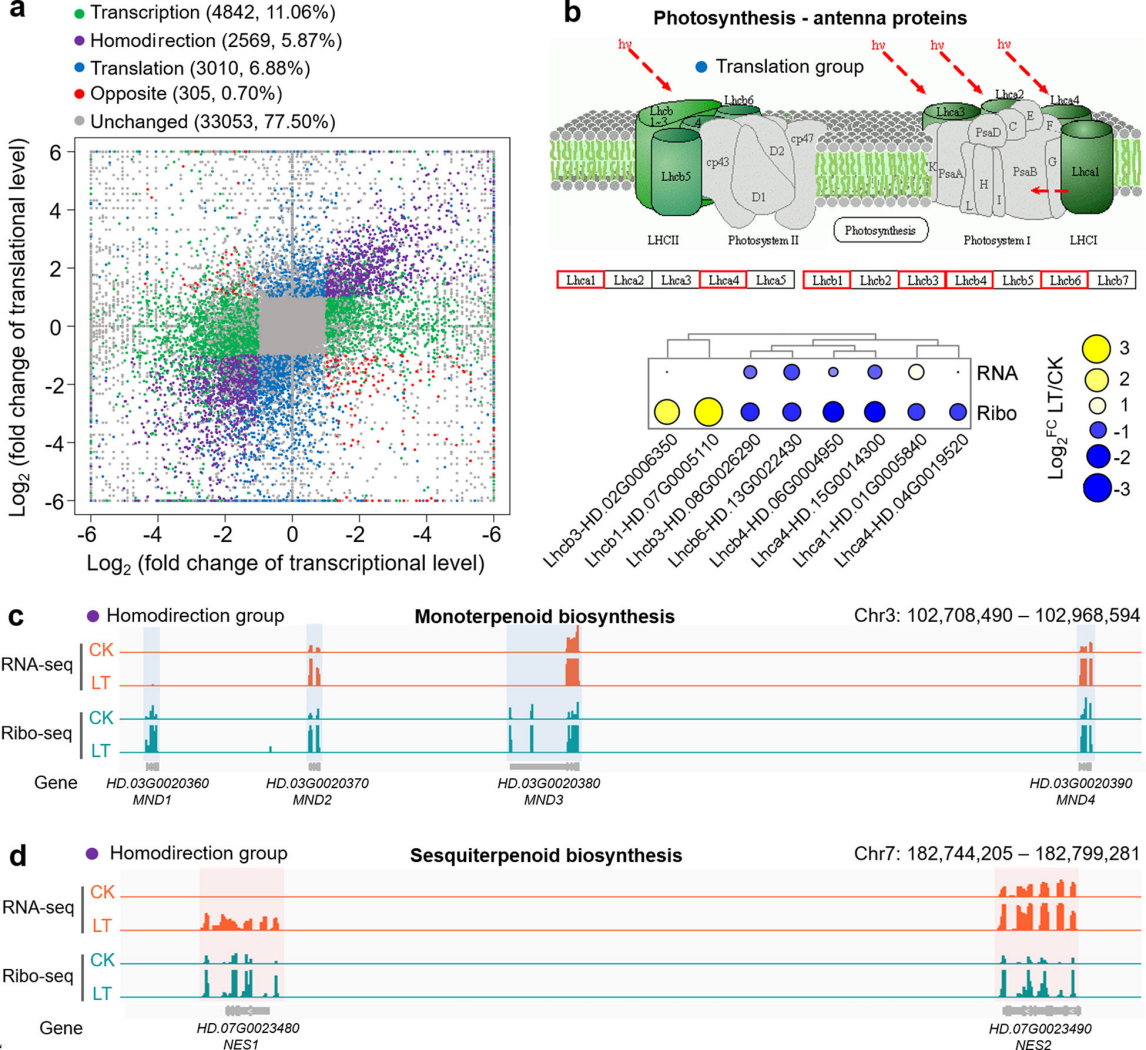
Transcriptional and translational changes in tea plant leave under chilling stress. **a** Scatter plot of fold changes at the transcription and translation levels under chilling stress. All genes were classified into five groups. **b** The genes in the translation group were significantly enriched in the photosynthesis-antenna protein pathway, and their transcription and translation levels are displayed in the form of heat maps. **c** Genes in the homodirectional group were significantly enriched in the monoterpenoid biosynthesis pathway. The IGV browser view shows that four (+)-neomenthol dehydrogenases (MNDs) were arranged in tandem on chromosome 3 and that their transcription and translation levels were significantly increased. **d** Genes in the homodirectional group were significantly enriched in the sesquiterpenoid biosynthesis pathway. The IGV browser view shows that two (*E*)-nerolidol synthases (NESs) were arranged in tandem on chromosome 7 and that their transcription and translation levels were significantly increased

To understand the effect of chilling stress on transcription and translation levels in tea plants in more detail, the genes that changed markedly at the transcription or translation level were defined by the criteria of fold change values ≥2 and false discovery rates (FDRs) below 0.05. We subdivided all genes into five groups (Fig. 3a), including a transcription group (11.06%, 4842 genes changed markedly only at the transcriptional level), a translation group (6.88%, 3010 genes changed markedly only at the translational level), a homodirectional group (5.87%, 2569 genes changed markedly at both the transcriptional and transactional levels and showed consistent trends), an opposite group (0.70%, 305 genes changed markedly at both the transcriptional and transactional levels and showed inconsistent trends), and an unchanged group (77.50%, 33,053 genes did not change markedly at either the transcriptional or transactional level).

We further performed a pathway enrichment analysis on the genes in the five groups (Fig. S7). Genes in the transcription group were significantly enriched in oxidative phosphorylation, carbon fixation in photosynthetic organisms, and glutathione metabolism pathways (Fig. S7). Genes in the translation group were enriched in the phenylpropanoid biosynthesis and photosynthesis-antenna protein pathways (Fig. S7). Among them, most genes in the photosynthesis-antenna protein pathway were significantly downregulated only at the translational level (Fig. 3b). Genes in the homodirectional group were significantly enriched in several pathways related to cold resistance, such as the plant–pathogen interaction, phenylpropanoid biosynthesis, plant hormone signal transduction, MAPK signaling, and monoterpenoid biosynthesis pathways (Fig. S7). Volatiles can mediate cold resistance in tea plants and trigger interplant communication^45,46^. Interestingly, we found that four (+)-neomenthol dehydrogenases (MNDs) in the monoterpene biosynthesis pathway were arranged in tandem on chromosome 3 (Fig. 3c), and two (*E*)-nerolidol synthases (NESs) in the sesquiterpene biosynthesis pathway were arranged in tandem on chromosome 7 (Fig. 3d) and that their transcription and translation levels were significantly increased. Genes in the opposite group were enriched in the photosynthesis, oxidative phosphorylation, and RNA polymerase pathways (Fig. S7).

### Chilling stress altered the TE of genes

Translational efficiency (TE) is a key indicator of RNA utilization efficiency and is calculated as FPKM_Ribo-seq_/FPKM_RNA‐seq_^31^. We attempted to evaluate whether tea plants can respond to chilling stress through altered TE in their genes. Our results showed that the median TE of LT samples was lower than that of CK samples (Fig. S8a), indicating that chilling stress also inhibited translation efficiency in tea plants. A total of 2287 genes with increased TE and 2686 genes with decreased TE under chilling stress were detected (Fig. S8b), and the correlation coefficients between the TE and transcription level of genes from the CK and LT samples were −0.38 and −0.37 (Fig. S8c, d), respectively. These results indicate that as the gene expression level increased, the TE of genes gradually decreased. Overall, the regulation of TE may be a chilling stress response method in tea plants.

### Identification of uORFs and their influence on the translation of mORFs

**Fig. 4:**
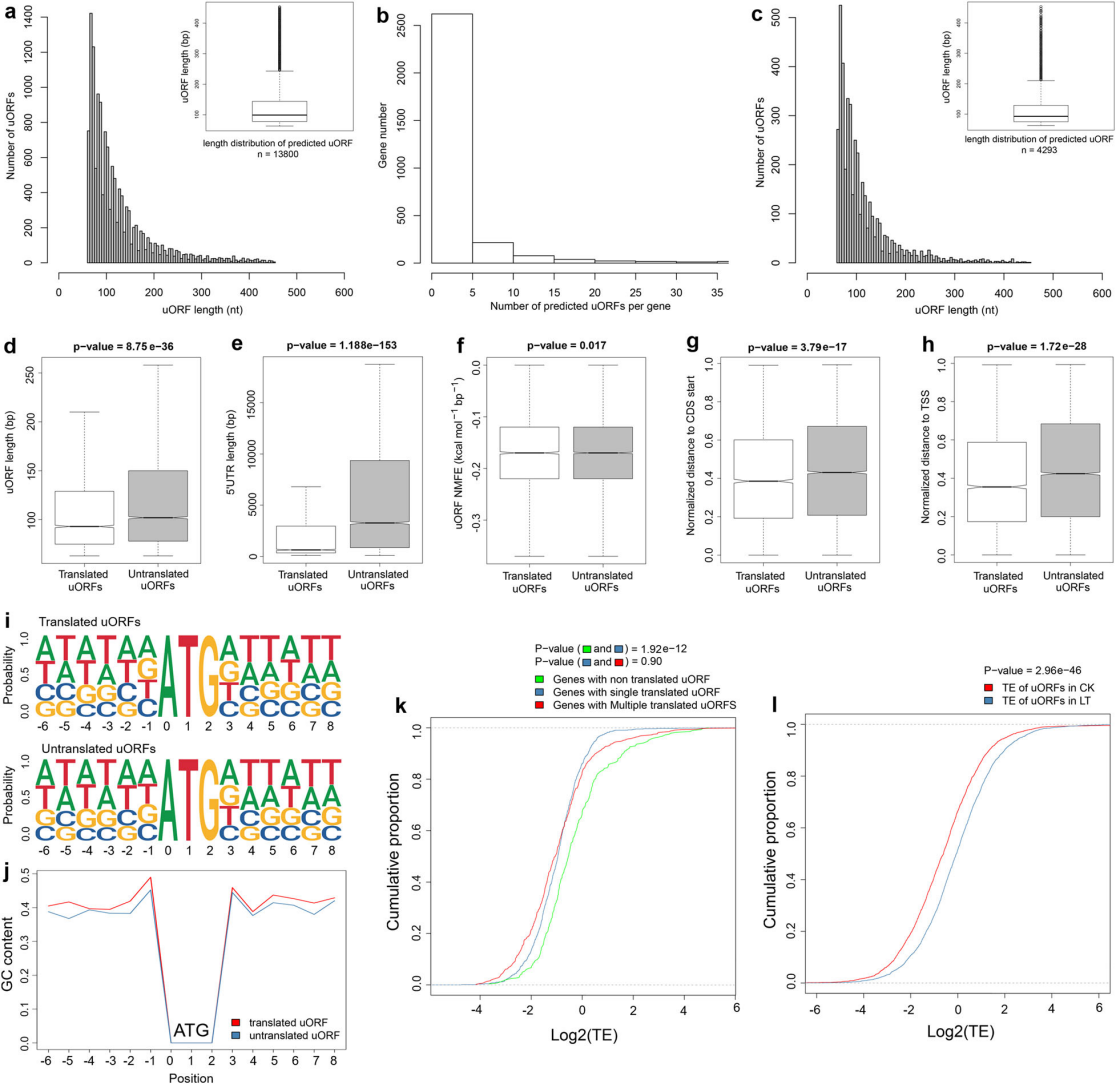
Identification and characteristics of upstream open reading frames (uORFs). **a** Length distribution of identified uORFs. **b** Number of identified uORFs in each gene. **c** Length distribution of the translated uORFs in the LT samples. **d**–**h** Comparison of translated and untranslated uORFs by uORF length (**d**), 5′ UTR length (**e**), uORF normalized minimal free energy (NMFE) (**f**), normalized distance to CDS start (**g**), and normalized distance to the transcription start site (TSS) (**h**) in the LT samples. *P* values were calculated by Student’s *t* test. **i** Sequence composition of translated and untranslated uORFs around the ATG start codon in the LT samples. **j** GC content of translated and untranslated uORFs around the ATG start codon in the LT samples. **k** Comparison of translational efficiency among genes with no, one, and multiple translated uORFs in the LT samples. The *P* value was calculated by the Kolmogorov–Smirnov test. **l** Comparison of translational efficiency of uORFs between CK and LT samples

Upstream open reading frames (uORFs), which are composed of a small open reading frame located in the 5′ UTR, can regulate the translation of downstream main ORFs (mORFs)^50^. However, there is still little information about uORFs in tea plants and their relationship with chilling stress. The Ribo-seq data made it possible to perform genome-wide uORF identification based on the ATG start codon in the 5′ UTR. Here, we identified 13,800 uORFs in the 5′ UTR of 3082 genes in tea plants (Fig. 4a), of which 2621 (85.04%) genes had 1–5 uORFs (Fig. 4b). We found 4031 and 4293 translated uORFs (FPKM ≥ 1) in the CK (Fig. S9a) and LT samples (Fig. 4c), respectively.

To compare the characteristics of translated and untranslated uORFs, we analyzed three indicators related to mORF translation, including the uORF length, 5′ UTR length, and normalized minimal free energy (NMFE). Compared with findings from the monocots rice^51^ and maize^28^, the length of the translated uORFs (Fig. 4d) and the 5′ UTR (Fig. 4e) of genes with translated uORFs were shorter than those in untranslated uORFs in tea plants, and they all showed the same folding potential (Fig. 4f). In addition, the relative distances from the uORF to the mORF start codon (Fig. 4g) and the transcription start site (TSS, Fig. 4h) were shorter in the translated uORFs. The differences in these sequence features were consistent with those in the CK samples (Fig. S9b–d). We further evaluated the frequency of bases surrounding the uORF start codon between translated and untranslated regions, as this codon is critical for translation initiation^52,53^. The probability of guanine at the −1 position was higher in the translated uORFs than in the untranslated uORFs (Fig. 4i), and a higher GC content in the translated uORFs was observed (Fig. 4j).

To explore the impact of translated uORFs on the TE of genes in tea plants, we focused on analyzing the TE of three sets of genes, including genes with a single translated uORF, genes with multiple translated uORFs, and genes with untranslated uORFs. Obviously, compared to untranslated uORFs, translated uORFs were associated with a significant reduction in the TE of genes. However, the number of translated uORFs did not significantly affect the TE of the genes (Fig. 4k).

Since the number of RFs in the 5′ UTR increased under chilling stress, the TE of uORFs also changed (Fig. 2b). We calculated the TE of the translated uORFs and observed that the TE in the LT samples was significantly higher than that in the CK samples (Fig. 4l), indicating that the translation of uORFs in tea plants was markedly enhanced under chilling stress.

### Features of ACRs under chilling stress

**Fig. 5:**
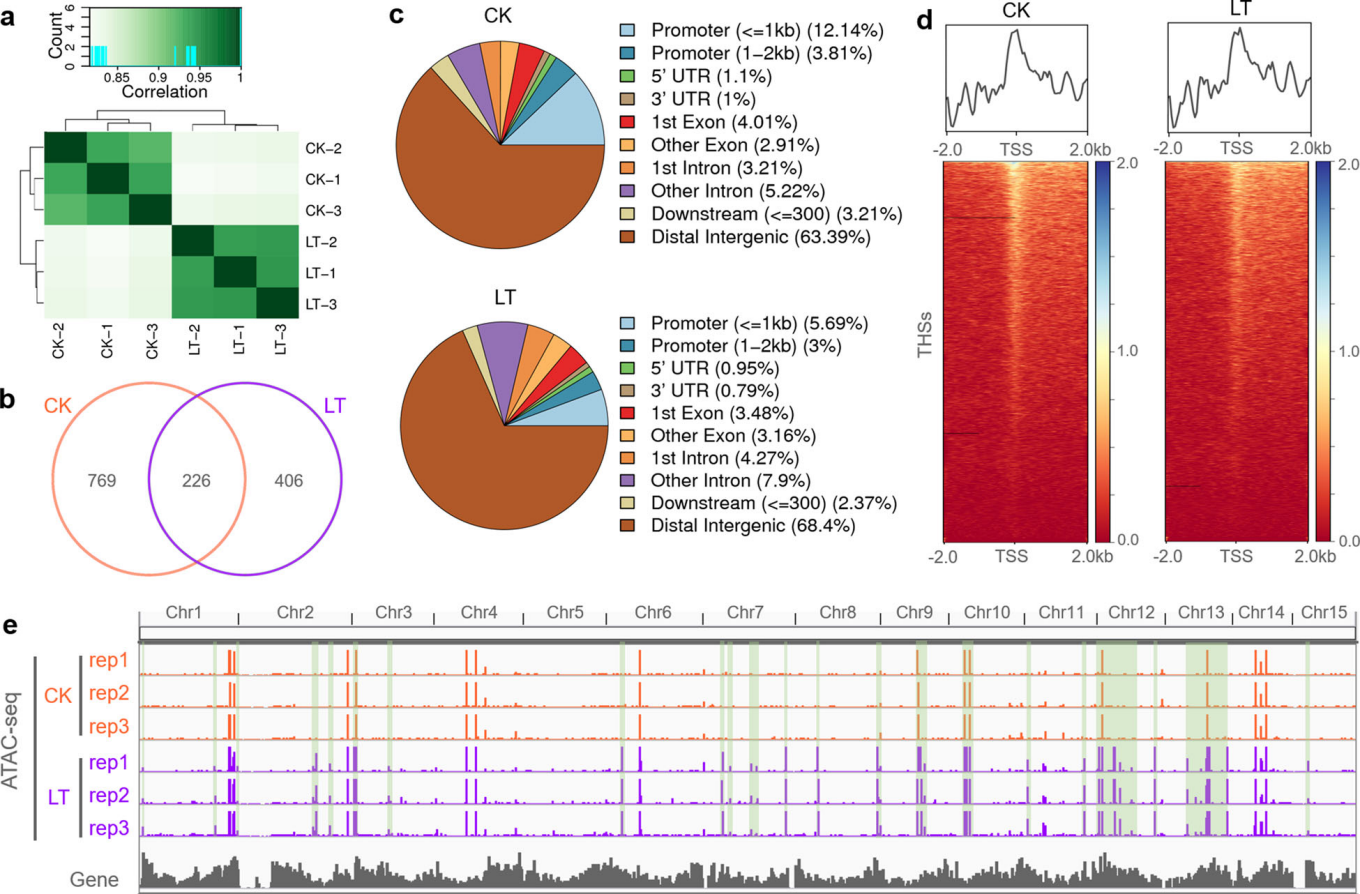
Chromatin accessibility landscape of CK and LT samples. **a** Heatmap showing the Pearson correlation coefficient of three ATAC‐seq replicates of two group samples. **b** Venn diagram showing the shared THSs between two ATAC-seq data sets. **c** Genomic distributions of THSs from CK and LT. **d** Average plots and heat maps showing the signals at the THSs in the ATAC-seq data sets. **e** IGV browser view showing the global ATAC-seq tracks of CK and LT. The areas of significantly increased chromatin accessibility in the LT samples are highlighted in light green shading

ATAC-seq is an effective method for obtaining many highly reproducible reads of ACRs^18^. The ATAC-seq experiment was performed in three biological replicates for CK and LT, and the results were highly reproducible (Fig. 5a). Only the THS regions that could be identified in all biological replicates will be further analyzed. Here, 997 reproducible THSs were identified in the CK sample, and 633 were identified in the LT sample, of which 226 THSs overlapped (Fig. 5b). The THSs from the two samples showed a similar distribution pattern in the tea plant genome; THSs distributed in the distal intergenic regions accounted for the majority, those in the promoter regions 2 kb upstream of the gene TSSs were the next largest group, and the exon and intron regions had the lowest numbers (Fig. 5c). The average plots and heatmaps of chromatin signals from two samples were examined (Fig. 5d), and the results showed that these signals tended to be enriched in TSS regions, as previously described in many plants^26^. The ATAC-seq data sets of the CK and LT samples were visualized in the IGV browser (Fig. 5e), and the high consistency between the three biological replicates further demonstrates the reliability of our data. It is important to note that the chromatin landscapes of the LT samples were significantly different from those of the CK samples, indicating chromatin regulation in tea plants under chilling stress.

### Analysis of distal accessible chromatin and related genes

CREs in distal accessible chromatin regions (dACRs) may play an important role in the long-range transcriptional control of genes in plant genomes^20,21^. Thus, we further analyzed the 433 distal THSs enriched in the LT sample. These distal THSs were mapped to 318 of the nearest annotated genes (Table S4), of which 275 distal THSs were more than 10 kb away from the nearest genes. We checked the expression and translation data for these 318 nearest annotated genes, and 71 (22.33%) showed significant changes at either the transcription or translation level or both. We further performed a KEGG pathway enrichment analysis on the 318 nearest genes, and 11 of them were significantly enriched in the oxidative phosphorylation pathway (Fig. S10). Respiratory adaptation is the compensatory adjustment mechanism of plants in response to chilling stress, and the oxidative phosphorylation of plant mitochondria is involved in respiratory adaptation to cold stress^54,55^. Our results imply that oxidative phosphorylation-related genes may be regulated remotely to adapt to low temperatures in tea plants. Previous evidence revealed that many distal THSs in maize may be potentially enriched with CREs^21^. Fisher’s exact test was performed to analyze the motif enrichment of distal THSs through MEME-AME^56^. We found that the enriched motifs were mainly related to three TF families, including 9 ERF-, 7 TCP-, and 6 WRKY-binding motifs (Table S5), which are involved in the plant stress response. Overall, our results showed that long-range transcriptional regulation may play an important role in the response of tea plants to chilling stress.

### Analysis of the genes associated with cold-induced THSs

**Fig. 6:**
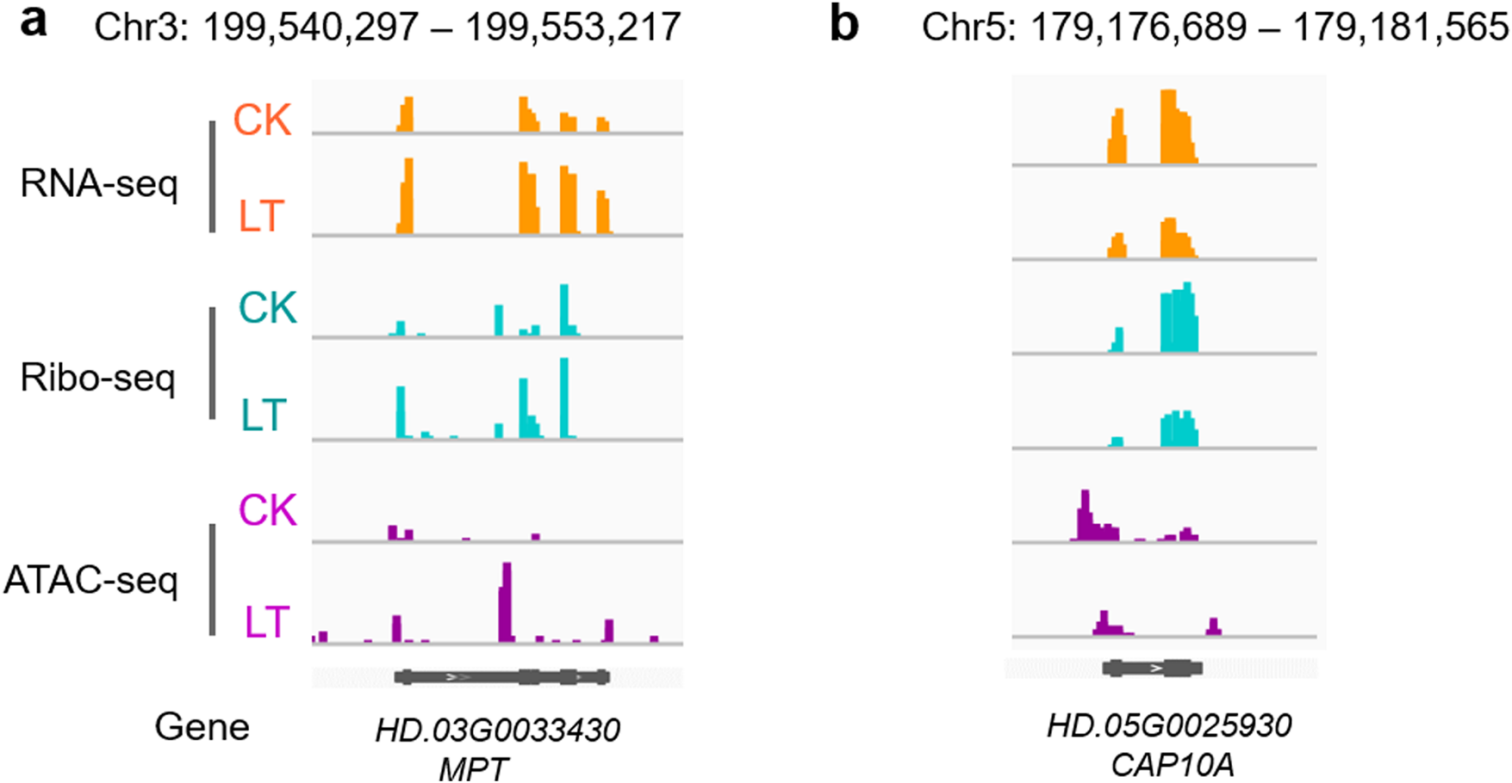
IGV browser views showing examples of significantly changed genes at the chromatin accessibility, transcription, and translation levels under chilling stress. **a** The mitochondrial phosphate carrier protein (*MPT*, *HD.03G0033430*) gene shows increased chromatin accessibility, transcription, and translation levels. **b** The chlorophyll a–b binding protein CP24 10A (*CAP10A*, *HD.05G0025930*) gene shows decreased chromatin accessibility, transcription, and translation levels

By evaluating the quantitative differences between the CK and LT samples in the THS data sets, excluding the distal THSs, we identified 66 cold-induced THSs (absolute fold change ≥2 and *P* < 0.05) that were mapped to 66 genes (Table S6). We found that 40.91% of the genes had significant changes at the transcription or translation level (Fig. 6). The relatively high frequency highlighted the potential role of cold-induced THSs in mediating cold regulation in tea plants. Pathway analysis of the annotated genes associated with cold-induced THSs showed that these genes were mainly related to fatty acid metabolism, ribosomes, photosynthesis-antenna proteins, and plant hormone signal transduction pathways (Fig. S11); these results overlap with the enrichment results for the DEGs determined via RNA-seq and Ribo-seq (Fig. S7).

### Motif identification in cold-induced THSs and construction of a transcriptional regulatory network

**Fig. 7:**
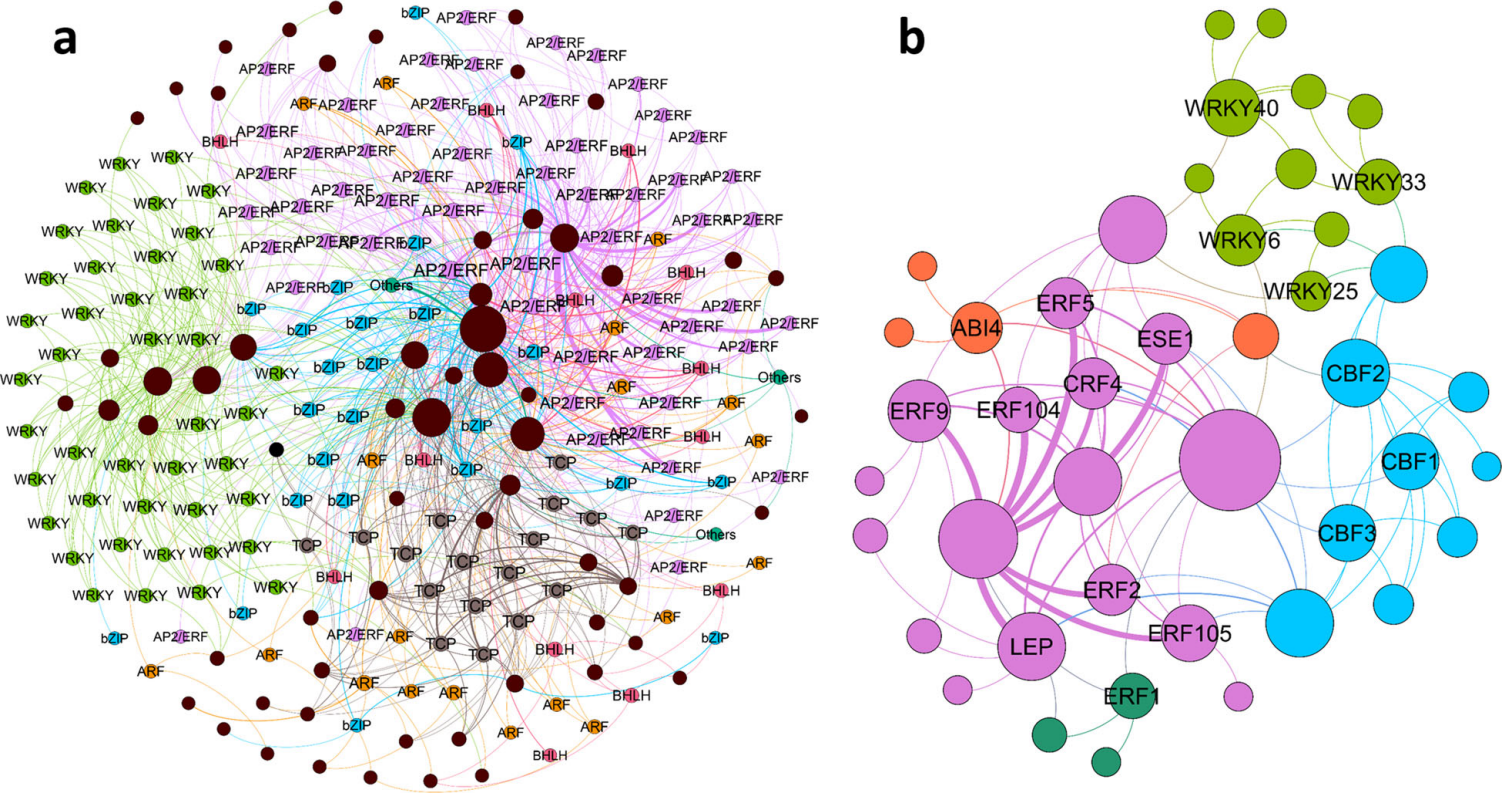
Transcriptional regulatory network of tea plants under chilling stress. **a** Regulatory network of TF families and cold-induced THSs. The round shapes represent TFs and targets, and the colored lines (edges) represent regulatory relationships. Different TF families are shown in different colors, and targets are shown in brown. Detailed information is provided in Table S7. **b** Regulatory network of 17 TF members and cold-induced THSs. The round shapes represent TFs and targets, and the colored lines (edges) represent regulatory relationships

Since cold-induced THSs represent more ACRs and may contain TF-binding motifs, we identified all cold-induced THSs with motifs through MEME. We found that the TFs that bind all identified motifs belong mainly to the AP2/ERF family, followed by the WRKY, bZIP, and TCP families (Fig. 7a and Table S7). Recent studies have also shown that the main regulatory TFs of three typical grass species under chilling stress are mainly ERFs^16^, which indicates the conservative regulatory network of plants under chilling stress. In addition, our results indicated the presence of a complex LT regulatory network in tea plants. For example, cold-induced THSs present in *HD.04G0004270* may be simultaneously regulated by TFs from the AP2/ERF, WRKY, TCP, bZIP, ARF, and bHLH families. We further selected 17 TF members that have been reported to function in plant responses to chilling stress to construct subregulatory networks (Fig. 7b and Table S7). These members belong to the AP2/ERF and WRKY families, including three CBFs that have been widely reported to improve plant cold tolerance^6,57^.

### Thirteen core TFs show significant changes in the transcription and translation levels of tea plants in response to cold

**Fig. 8:**
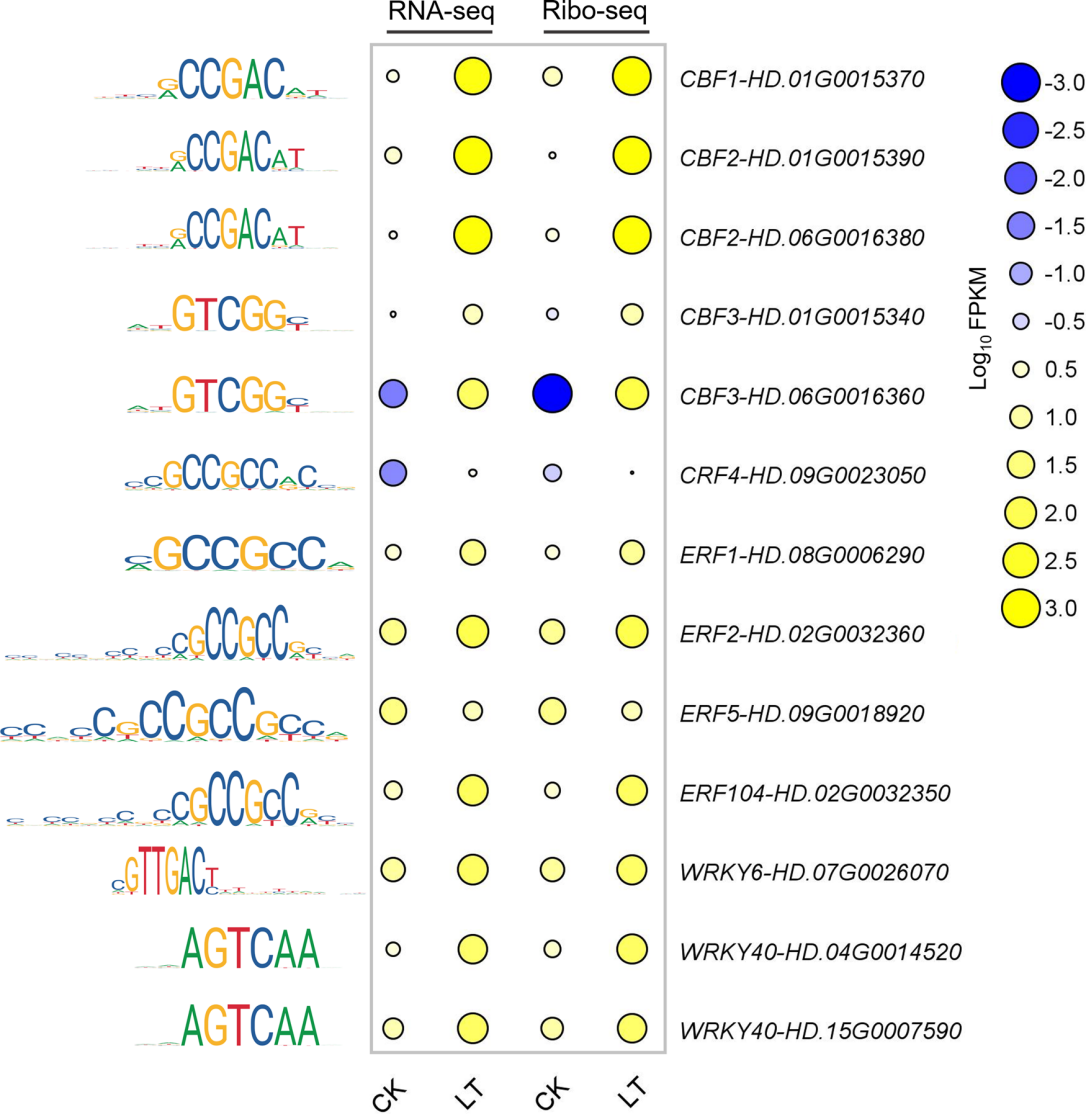
Thirteen core TFs show significant changes at the transcription and translation levels. The expression levels were generated according to the log10 normalization of the FPKM values calculated from three replicates. The size and color of each circle represent the expression level. On the left is the transcription factor binding motifs that have been verified in other plants and exist in cold-induced THSs. The position weight matrix describes the possibility of finding the corresponding nucleotide at each position in the motif

We were interested in whether the 17 important TFs revealed by ATAC-seq respond to chilling stress at the transcription and translation levels. Here, we identified 13 core transcription factors and found that the transcription and translation of 12 TFs were dramatically increased under chilling stress, while those of *ERF5* (*HD.09G0018920*) were downregulated by 4.61- and 3.88-fold, respectively (Fig. 8). As the core genes in the cold response pathways of other plants, the transcription and translation levels of the four CBF members (*CBF1*, *HD.01G0015370*; *CBF2*, *HD.01G0015390* and *HD.06G0016380*; *CBF3*, *HD.06G0016360*) increased several hundred100-fold, which was consistent with the results of previous studies on CBF genes in tea plants at the transcriptional level^47,58^ and indicated the conservation of *CBF* genes in the cold response. *ERF1*, *ERF2*, *ERF5*, *ERF104*, and *CRF4* are known regulators of low-temperature-responsive genes^16,59–61^ and may also play an important role in the chilling stress response in tea plants. WRKYs are considered to be an important regulator of plant environmental stimuli^62,63^, and they bind mainly to the W box in the promoter of the targets to regulate their expression^64^. Three WRKY members in tea plants (*WRKY6*, *HD.07G0026070*; *WRKY40*, *HD.04G0014520,* and *HD.15G0007590*) have been evaluated for their role in chromatin accessibility, transcription, and translation, and they may be involved in the regulation of chilling stress (Fig. 8). Thus, these 13 TFs may be the core regulators of the cold response in tea plants.

## Discussion

To date, several studies have focused on how chilling stress affects biological processes in plants at the mRNA level. However, the response of plants, especially tea plants, to chilling stress at the chromatin accessibility, transcription, and translation levels has not been investigated. In this study, we aimed to explore the cold-regulation landscapes of tea plant leaves at both the transcriptional and posttranscriptional levels by combining ATAC-seq, RNA-seq, and Ribo-seq technologies. These results can be expected to provide new insights into the responses and regulation of plants under chilling stress at multiple levels.

The translation process is highly dynamic, and the genetic information in mRNA molecules is converted into proteins that perform specific functions through this process^65^. However, the levels of mRNA and the corresponding protein are not always correlated. Thus, the responses to and regulation of the translation process should receive more research attention. Here, we observed that chilling stress inhibited both transcription and translation in tea plants (Fig. 2c, e) and found that the Pearson correlation coefficient between transcription and translation increased from 0.54 to 0.68 under cold stress (Fig. 2d). Similarly, relatively high Pearson correlations (*R*^2^ = 0.69) were also found in maize under progressive drought conditions^28^, indicating that plants can coordinate more effectively to respond to environmental stress. Translational modulation is a faster and more direct way of responding to environmental conditions and maintaining homeostasis^66^. There were 3010 genes that changed only at the translational level and were significantly enriched in the photosynthesis-antenna proteins and phenylpropanoid biosynthesis pathways (Fig. 3 and Fig. S7). Susceptibility to photoinhibition and the regulation of photosynthetic capacity are important processes of plant cold acclimation^67,68^. It has been reported in tea plants that the downregulation of photosynthesis-related genes may play an important role in cold tolerance^43^. We further discovered that the translation levels of some photosynthesis-related genes were independently inhibited under chilling stress. In addition, the regulation of phenylpropanoid biosynthesis is also a way for plants to resist cold^69,70^. These results indicate that photosynthesis and phenylpropanoid biosynthesis are crucial pathways involved in the rapid response to chilling stress at the translational level. We also focused on those genes whose expression was significantly altered at both levels and showed consistent trends. The enriched pathways of these genes were related to the cold response of plants, and they included phenylpropanoid biosynthesis^69,70^, plant hormone signal transduction^71^, and MAPK signaling^72,73^. Recent studies have revealed that volatiles can mediate the cold response of tea plants and trigger communication between plants^45,46^. Terpenoid volatiles, including nerolidol, linalool, and geraniol, can be emitted from tea plants at low temperatures^46^. Our results showed that the transcription and translation levels of four *MNDs* and two *NESs* arranged in tandem on the chromosomes increased markedly (Fig. 3c, d), which highlights the coordinated regulation of terpene synthase genes under chilling stress in tea plants. Overall, the regulation of transcription and translation at low temperatures is both relevant and independent, and this strategy increases the flexibility of gene expression and provides a molecular basis for adapting to changes in environmental temperatures and achieving coordinated regulation.

Consistent with the findings for hypoxic *Arabidopsis*^35^ and drought-stressed maize^28^, chilling stress has a negative impact on the TE of tea leaves (Fig. S8). The significant decrease in TE may be due to the reduced ribosome occupancy of the initiation codon, resulting in reduced translation initiation^65^. uORFs also affect the translation of corresponding genes and mainly play an inhibitory role^33^. Our results showed that the translated uORFs significantly reduced the TE of the corresponding genes (Fig. 4k). However, inconsistent with previous studies^28,74^, an increased number of translated uORFs did not increase the suppressive effect. Furthermore, the number of RFs in the 5′ UTR in the LT samples increased, and the TE of the translated uORFs also increased significantly (Fig. 4l). These results revealed the mechanism by which tea plants inhibit the overall expression of genes by enhancing the translation of uORFs under chilling stress. We compared various features of translated and untranslated uORFs (Fig. 4d–j), and their differences were closely related to initiation codon recognition in uORFs and translation initiation by mORFs^49,51,75^. Recent reports have shown that changing the translation of mRNA by regulating uORFs can effectively improve crop qualities^76–78^. For instance, editing the uORF of a key gene related to vitamin C biosynthesis in lettuce effectively improved its antioxidant capacity and ascorbate content^78^. Therefore, the uORFs identified by ribosome profiling in this study provide a new possibility for improving cold tolerance in plants.

The chromatin accessibility landscape under a specific condition is considered to represent the range of protein binding sites under this condition, which helps us to further explore transcriptional regulatory events in the whole genome^9^. Cold conditions enhance chromatin accessibility and bivalent histone modification in active genes in potatoes, forming a distinct chromatin environment^79^. Our research showed that the chromatin landscape of tea plants changed significantly at low temperatures (Fig. 5e). Interestingly, the majority of THSs were located in distal intergenic regions (Fig. 5c), indicating that long-range transcriptional regulation may play an important role in the response of tea plants to chilling stress. However, this distribution is different from those in many plants^20,26^, possibly due to the large number of repetitive sequences (80%~) in the tea plant genomes^80,81^. A comparison of ATAC-seq data from 13 plant species suggested that most of the distal THSs originated from the proximal region of the genes and became distal to the genes due to the proliferation of transposable elements and showed that distal THSs were preferentially located upstream and adjacent to their target genes^20,21^. Thus, we preliminarily defined the closest downstream gene as the target gene of the distal THS and found that the target genes were related to oxidative phosphorylation, which is involved in a respiratory adaptation by plants to cold^54,55^. We also observed that many CREs were enriched in the cold-induced distal THS, including 9 ERFs, 7 TCPs, and 6 WRKYs. These results provide indications of long-range transcriptional regulation in plants under chilling stress.

Several studies have used well-characterized motifs on THSs to construct transcriptional regulatory networks^22,24–26^, indicating that it is feasible to infer regulatory networks based on ATAC-seq results. In fact, the findings from our study (Fig. 7) and another recent study on three typical grasses^16^ revealed that ERFs were the common regulatory factors in plants under chilling stress; these findings indicate the conservation of regulatory TFs across plant species. We also identified 13 crucial TFs indicating the roles of chromatin, transcription, and translation under chilling stress (Fig. 8); these TFs include CBFs that have been revealed to play a key role in tea plants^47,82,83^ and other plants^57,84^ as well as ERF2^16^, CRF4^61^, and 3 WRKYs^62^. These high-confidence regulatory TFs demonstrate the reliability of our results and can help to elucidate the regulatory relationships that may CK cold-responsive gene expression.

## Materials and methods

### Plant materials and treatments

Two-year-old potted tea plants (cv. Fudingdabaicha) planted on the tea plantation of Fujian Agriculture and Forestry University (Fuzhou, China; 26°08′19″ N, 119°24′06″ E) were transferred to a climate-controlled chamber (23 °C, 60% humidity) with a 16/8 h (light/dark) photoperiod. After 15 days, half of the tea plants were transferred to a different chamber set at 4 °C with the same humidity and photoperiod for 24 h. Three biological replicates were obtained from the many individual tea plants in the CK and LT treatments, and their second and third leaves were collected and frozen in liquid nitrogen for further analysis.

### Processing and analysis of RNA-seq data

The RNA-seq data were processed and analyzed as described in our previous research, with minor modifications^85^. Briefly, the total RNA from CK and LT was used to construct a library and sequenced using an Illumina HiSeq2500. Subsequently, the raw reads were filtered and mapped to the newly released chromosome-level tea plant genome (https://bigd.big.ac.cn/search/?dbId=&q=CRA003208) with the HISAT2 program^86^. StringTie v1.3.1 was used to calculate the fragment per kilobase of transcript per million mapped reads (FPKM) value^87^. Differentially expressed gene (DEG) analysis was performed using DESeq2^88^. Genes with absolute fold change values ≥2 and FDRs below 0.05 were considered DEGs. We further used ClusterProfiler^89^ to perform GO and KEGG enrichment analyses on the DEGs and visualized the results on the OmicShare website (http://www.omicshare.com/tools).

### Quantitative real-time PCR (qRT-PCR)

cDNA synthesis and qRT-PCR were performed to test the reliability of the transcriptome data based on a previously described method^90^. Primers for 12 randomly selected DEGs were designed and verified by Primer3Plus (http://www.bioinformatics.nl/cgi-bin/primer3plus/primer3plus.cgi). The primer information is listed in Table S8, and the *CsGAPDH* (GE651107) gene was used as a reference control. The sample was analyzed in three biological replicates, and the relative expression values were obtained using the 2^−△△Ct^ method^91^.

### Ribosome profiling and sequencing

The methods of ribosome profiling and sequencing were consistent with those used in previous experiments successfully performed on tea plants^30^. Briefly, three replicate samples from the CK and LT treatments were ground in liquid nitrogen and dissolved in a lysis buffer. The lysates were centrifuged, and the supernatants were collected for RF preparation. Five microliters of DNase I and 7.5 µL of RNase I were added to 300 µL of lysate and incubated for 45 min, and the reaction was stopped by adding SUPERase·In RNase inhibitor. Subsequently, the size exclusion column (Illustra MicroSpin S-400 HR Columns, GE Healthcare) was equilibrated with 3 mL of polysome buffer by gravity flow and centrifuged. RFs larger than 17 nt were isolated by an RNA Clean and Concentrator-25 kit (Zymo Research, Beijing, China). After removing the rRNA, the RFs were purified, and the library was constructed. Sequencing was performed by Gene Denovo Biotechnology (Guangzhou, China) on an Illumina HiSeqTM 2500.

### Ribo-seq data analysis

Low-quality data filtering and rRNA data removal were performed on the raw data obtained by Ribo-seq. The remaining clean reads were mapped to the chromosome-level tea plant genome (https://bigd.big.ac.cn/search/?dbId=&q=CRA003208) by Bowtie2^92^, and the genome features of RFs were analyzed. The RF density was calculated to verify the reliability of Ribo-seq. RiboTaper^93^ was used to calculate the read number in the ORF of coding genes and calculate their FPKM value. The edgeR package^94^ was run to identify genes that were differentially expressed at the translational level. Genes with absolute fold change values ≥ 2 and FDRs below 0.05 were considered DEGs at the translational level. The NMFE was used to identify the sequence stability of the secondary structure, which was calculated by RNAfold and normalized to the sequence length.

### Combined Ribo-seq and RNA-seq data analysis

Based on the expression changes at the translational and transcriptional levels, we divided the genes into five groups, including a transcription group (changed markedly only at the transcriptional level), a translation group (changed markedly only at the translational level), a homodirectional group (changed markedly at two levels and showed consistent trends), an opposite group (changed markedly at two levels and showed inconsistent trends), and an unchanged group (did not change markedly at either level). Genes from the above groups were analyzed for their GO functions and KEGG pathway enrichment. Heatmaps of expression at the two levels were generated using TBtools^95^. The TE is the ratio of translated mRNAs to the total mRNAs per gene, and it was calculated as FPKM_Ribo-seq_/FPKM_RNA‐seq_.

### Analysis of uORFs

The sequence in the 5′ UTR of known protein-coding genes was extracted to identify uORFs with a length ranging from 60 to 450 nt and containing the ATG start codon. The translated uORFs were defined by FPKM ≥ 1. The SeqLogo R package was used for motif analysis around the ATG start codon between translated and untranslated uORFs.

### Library preparation and THS identification of ATAC-Seq

According to the standard ATAC-seq protocols^1^, we lysed the cells to obtain the nuclear suspension and incubated them in a transposition mix that included a transposase. The transposition reaction was incubated at 37 °C for 30 min. After transposition, the products were purified and amplified according to the QIAGEN MiniElute kit method and sequenced using Illumina HiSeqTM 4000 by Gene Denovo Biotechnology (Guangzhou, China). Three biological replicates were prepared for CK and LT.

After filtering, we used the Bowtie2 program^92^ to map the clean reads to the latest chromosome-level tea plant genome (https://bigd.big.ac.cn/search/?dbId=&q=CRA003208) and filtered the reads aligned with mitochondria or chloroplasts. Samtools^96^ was used to convert sam format files to bam files and to sort the data. Peak calling was performed on MACS 2.1.2^97^ with the parameters “-nomodel -shift −100 -extsize 200 -B -q 0.05”, and the resulting peaks were considered transposase hypersensitive sites (THSs). The signal distribution of ATAC-seq data was analyzed and visualized with DeepTools^98^. Integrative Genomics Viewer (IGV) 2.8.3^99^ was run to visualize the bigwig file of ATAC-seq data. ChIPseeker 1.16.1^100^ was used to confirm the genes related to THSs and analyze the distribution of THSs in different genomic regions. GO and KEGG enrichment of the THS-related genes was analyzed by clusterProfiler^89^. The R package DiffBind was used to analyze the differences in THS between the CK and LT groups. THSs occurred only in cold-treated samples and with absolute fold change values ≥ 2, and a value of *P* < 0.05 indicated a cold-induced THS in tea plants.

### TF motif calling and regulatory network construction

THSs shared by three replicates of CK and LT were subjected to motif analysis. MEME-ChIP^101^ was used for motif identification from the THS sequences (*E* value < 0.05), and functional annotation of the motifs was performed with the AME^56^, *Cis*-BP^102^, and DAP-seq^103^ databases. FIMO^104^ was used to identify motif occurrences for TFs, and the obtained TFs and target genes were used to construct a transcriptional regulatory network using Gephi 0.9.2^105^.

## Supplementary information

Figure S1-S11

Table S1-S8

## Data Availability

The raw ATAC-seq data (Accession no. CRA003567), raw Ribo-seq data (Accession no. CRA003570), and raw RNA-seq data (Accession no. CRA003568) were uploaded to the National Genomics Data Center (https://bigd.big.ac.cn/) under Project no. PRJCA003963.
