# Supplementary material for: Chromatin accessibility and translational landscapes of tea plants under chilling stress
Source: Hortic Res. 2021 May 1;8:96. doi: 10.1038/s41438-021-00529-8 (PMC8087716; doi:10.1038/s41438-021-00529-8)
Supplement: Supplementary file 1 — Figure S1-S11 [file 41438_2021_529_MOESM1_ESM.docx]

**
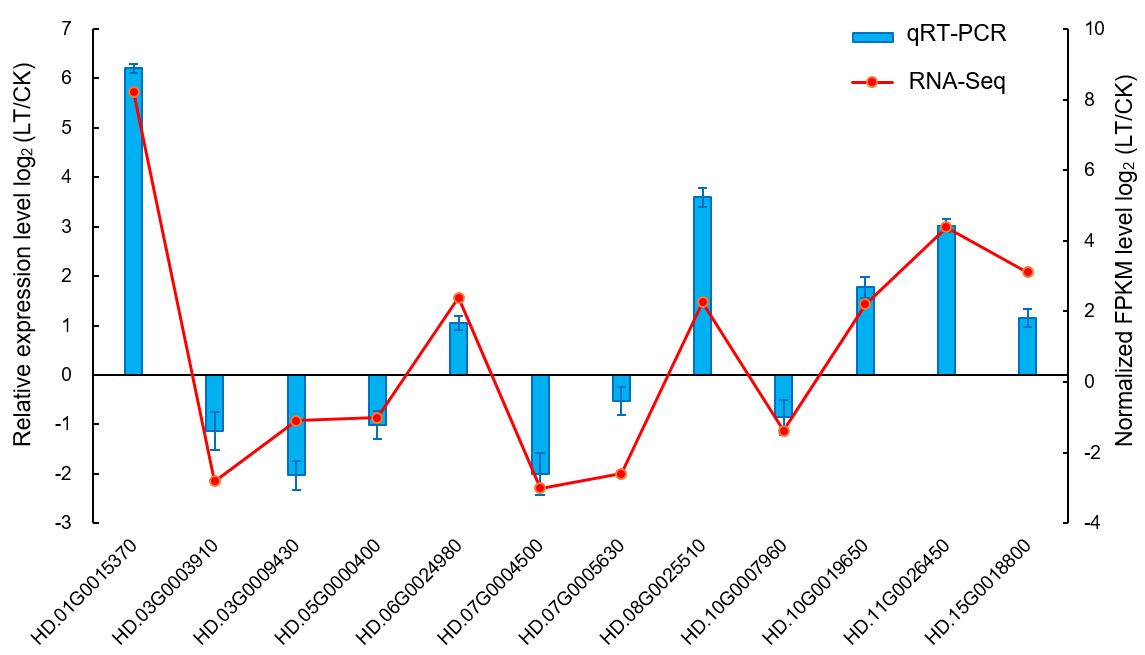
**

**Figure S1. Validation of the transcription levels for randomly selected DEGs by qRT-PCR.**


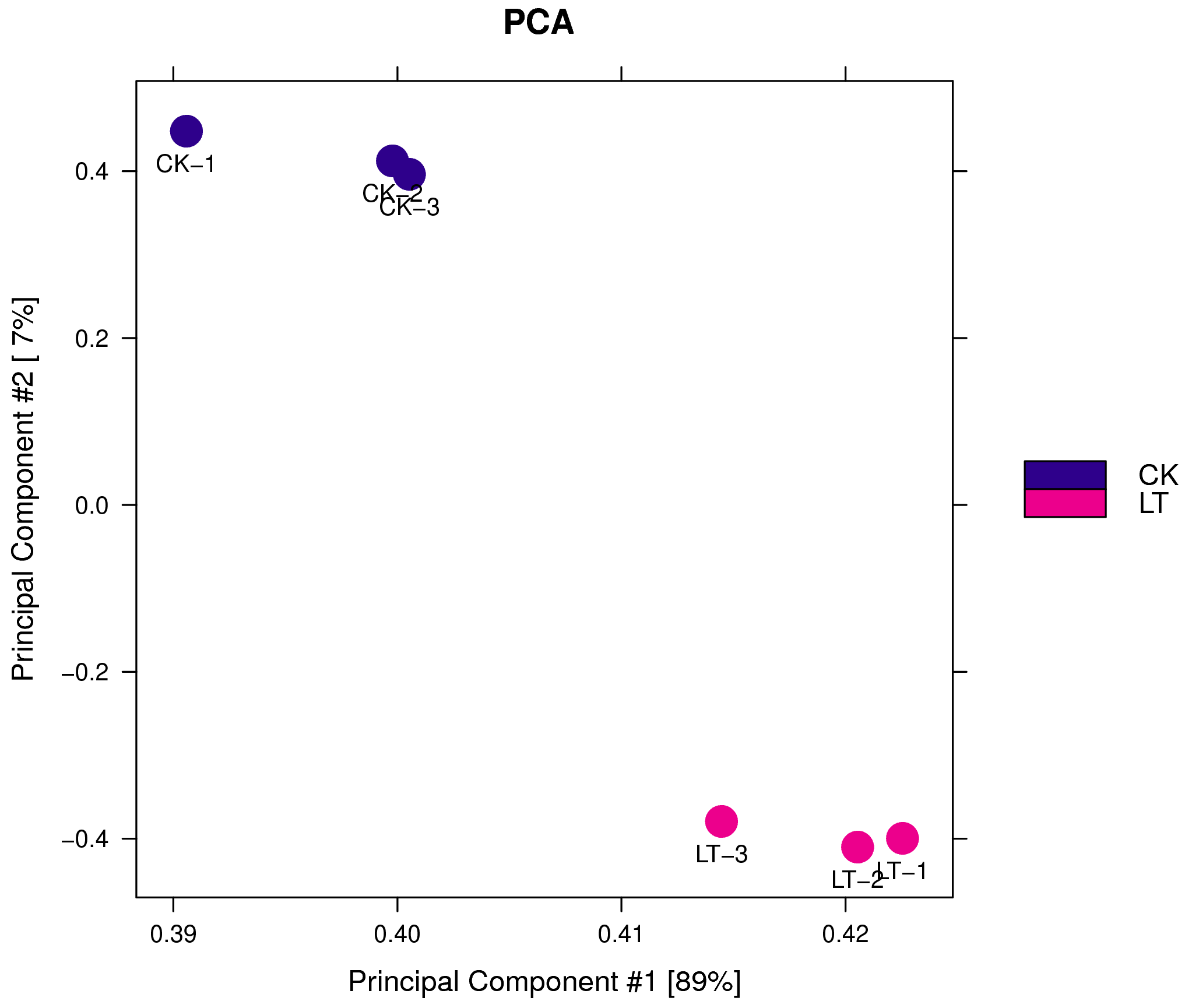


**Figure S2. Principal component analysis of ATAC-seq data of CK and LT sample**


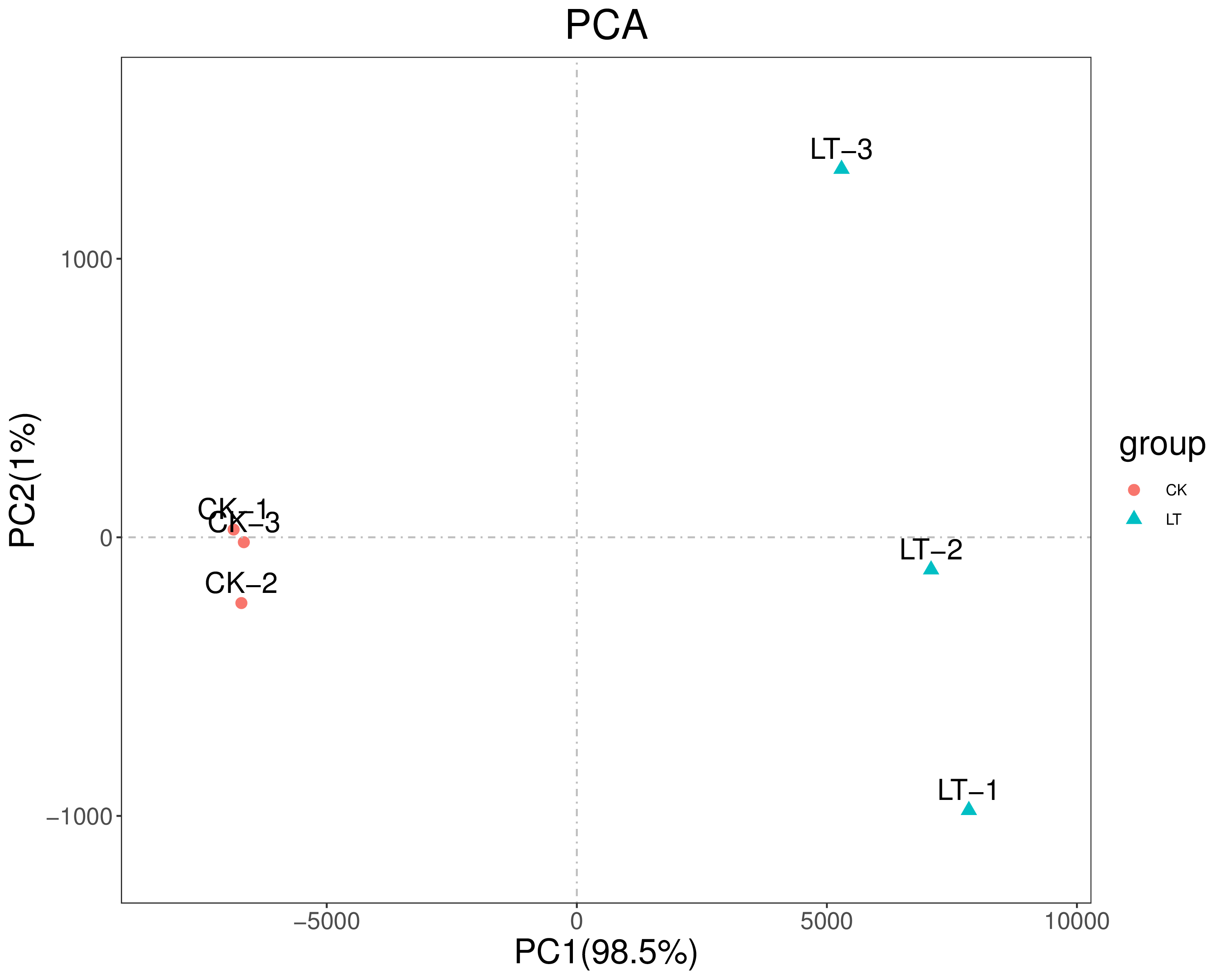


**Figure S3. Principal component analysis of RNA-seq data of CK and LT samples**


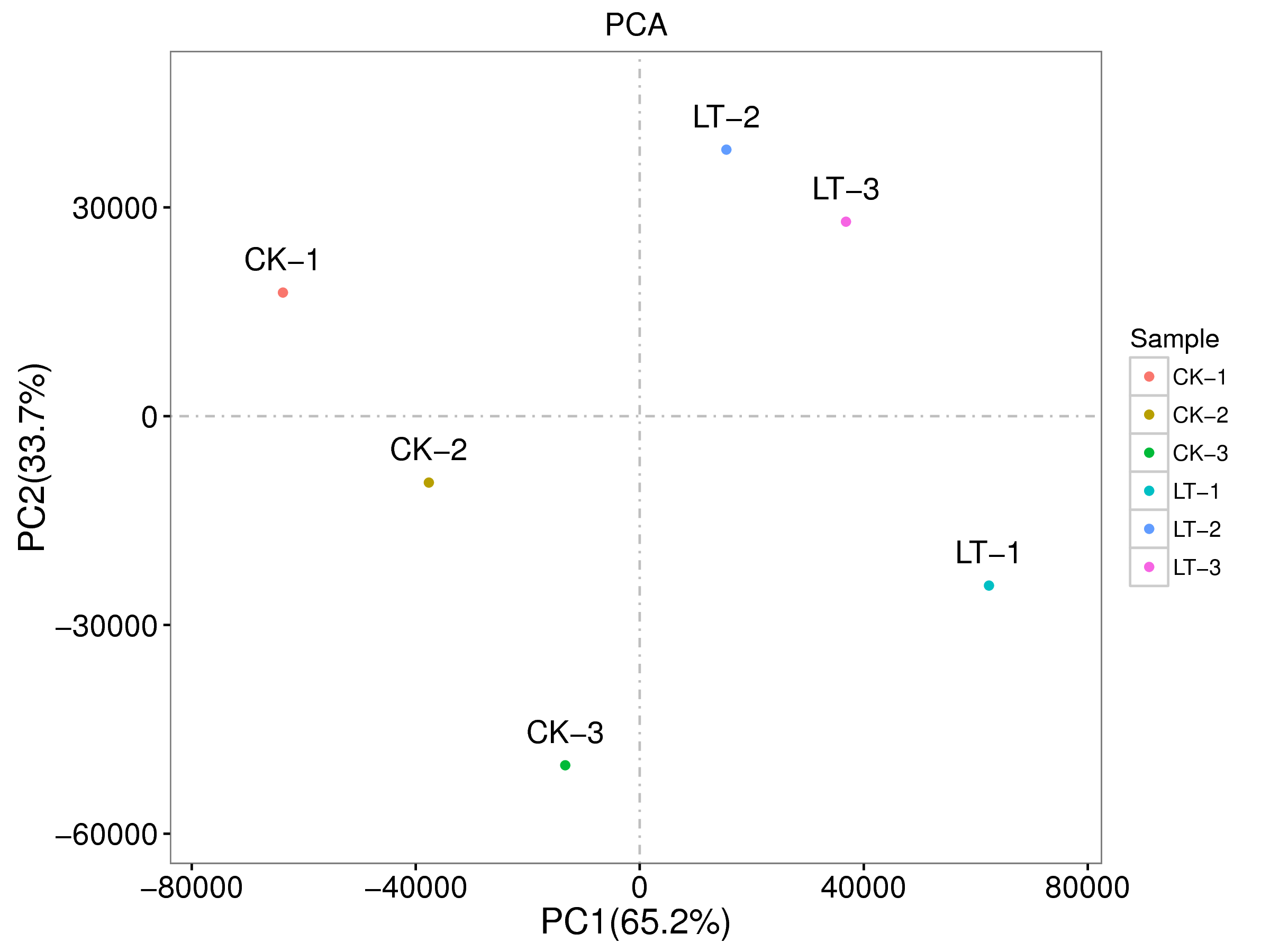


**Figure S4. Principal component analysis of Ribo-seq data of CK and LT samples**


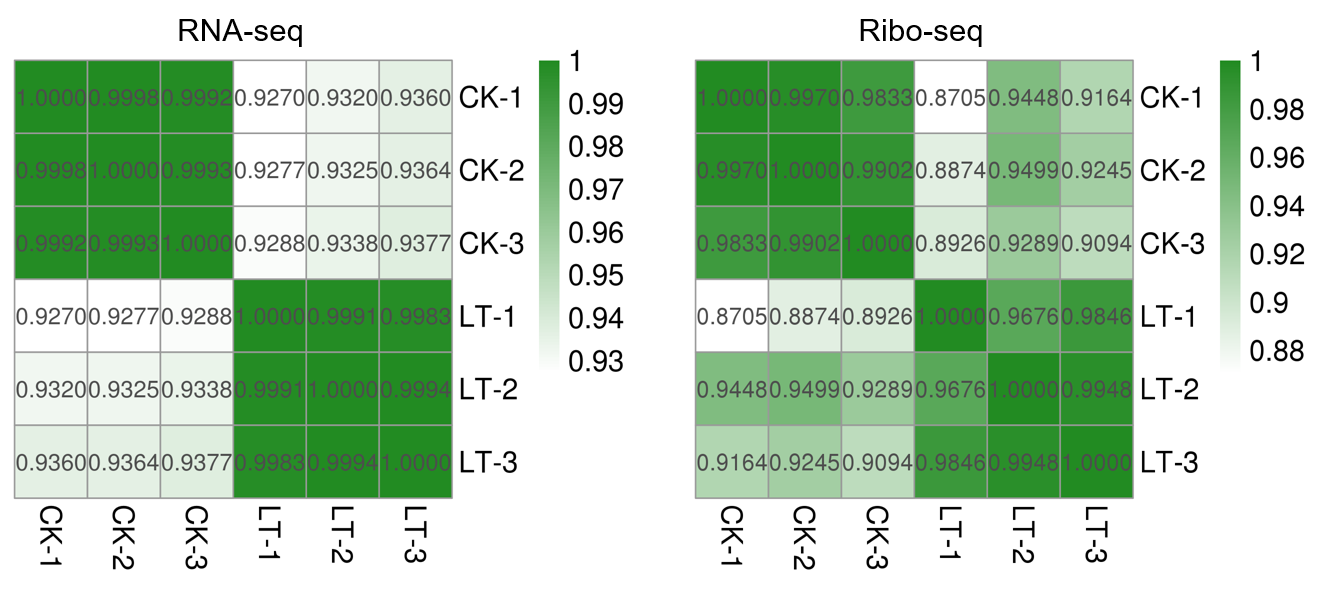


**Figure S5. Correlation analysis of RNA-seq and Ribo-seq data of CK and LT samples**


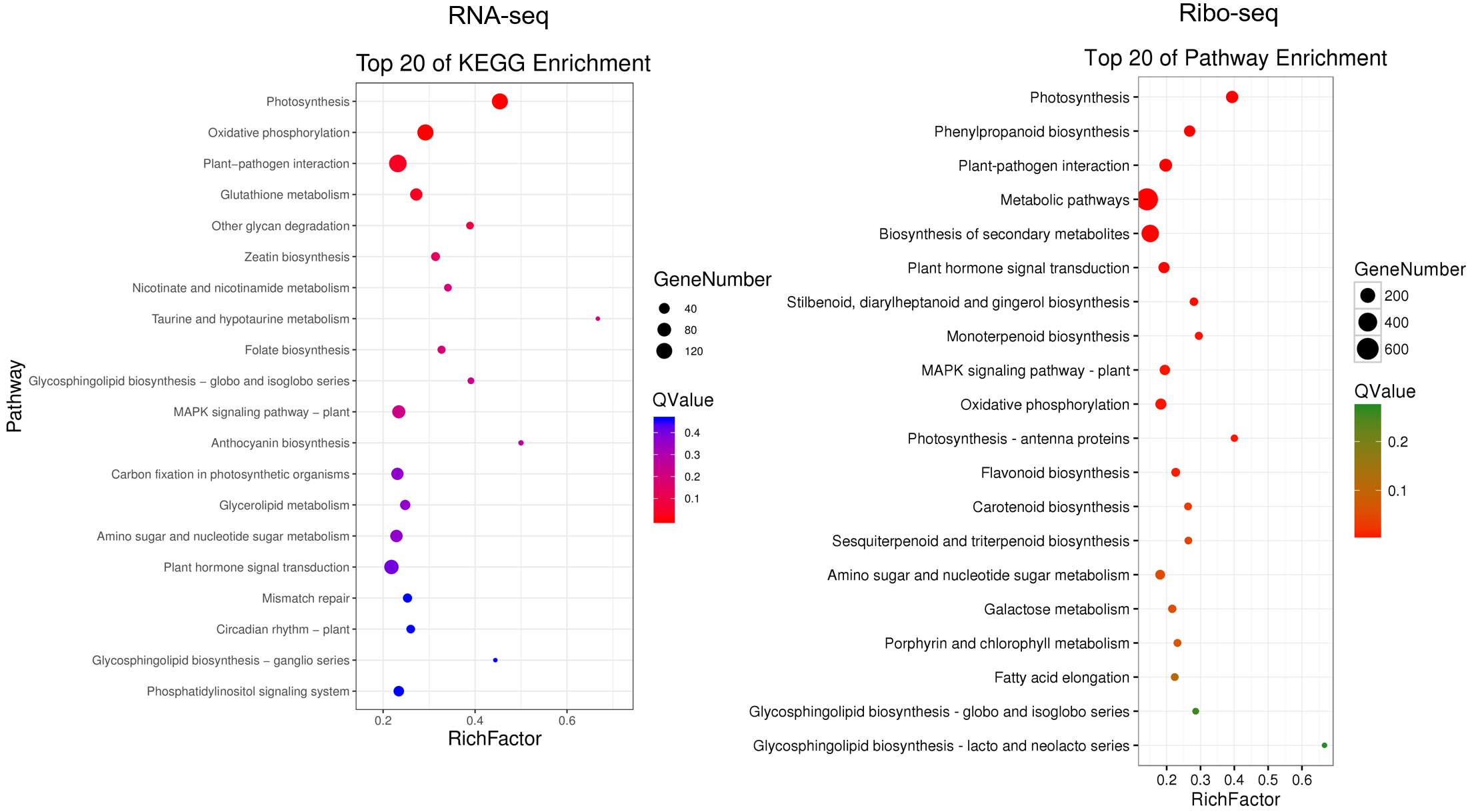


**Figure S6. Top 20 KEGG pathways of DEGs at transcriptional and translational levels**


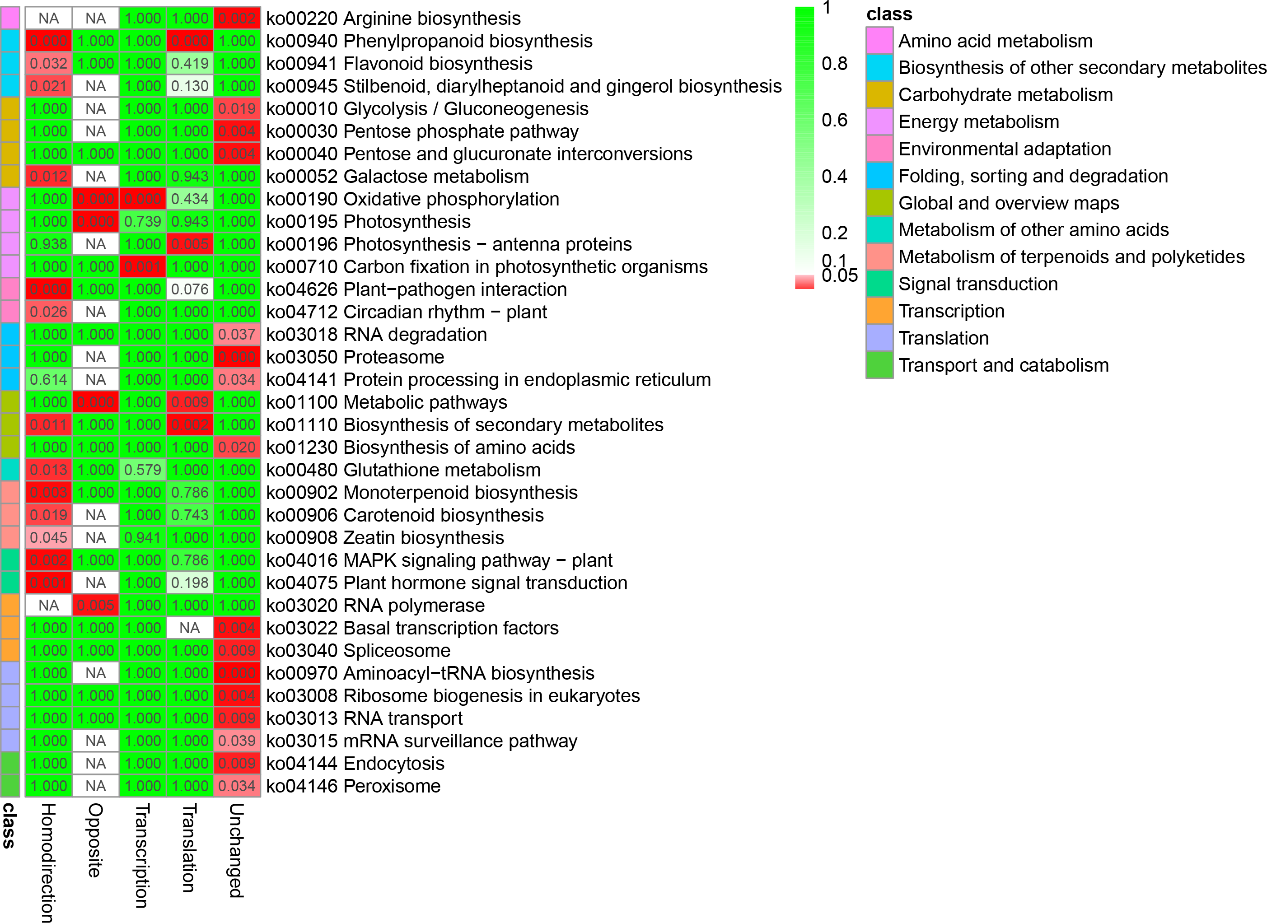


**Figure S7. KEGG pathway analysis of genes in five groups**


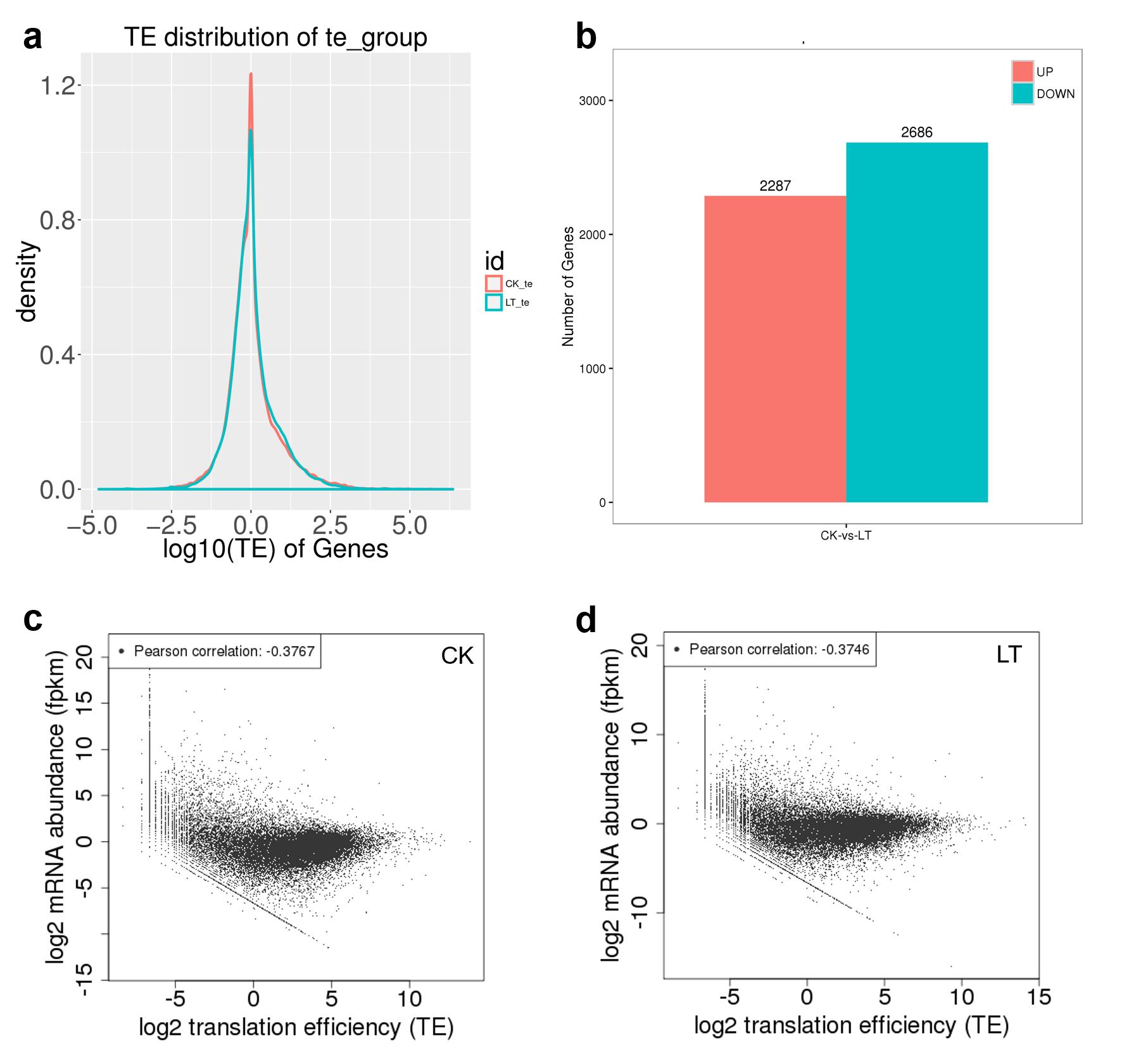


**Figure S8. The features of translational efficiency under low temperatures. a** Distribution of TE between CK and LT samples. **b** Statistics of genes that change significantly at translational efficiency levels. **c** Pearson correlation coefficient between translational efficiency and transcriptional level from CK samples. **d** Pearson correlation coefficient between translational efficiency and transcriptional level from LT samples.


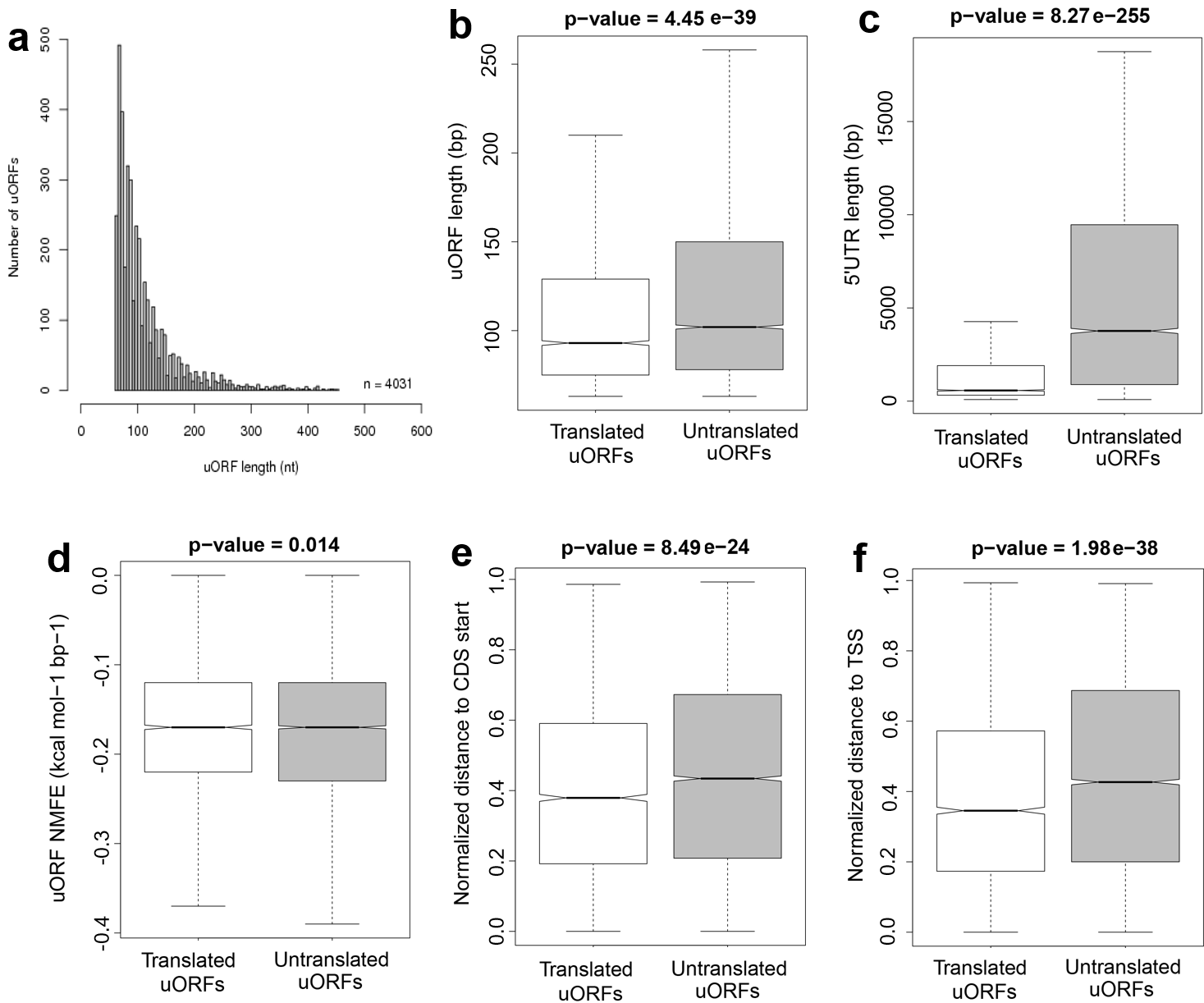


**Figure S9. Identification and characteristics of upstream open reading frames (uORFs) in CK samples. a** The length distribution of the translated uORFs in the CK samples. **b-f** Comparion of translated and untranslated uORFs in uORF length (**b**), 5′ UTR length (**c**), uORF normalized minimal free energy (NMFE) (**d**), normalized distance to CDS start (**e**), and normalized distance to transcription start site (TSS) (**f**) in the CK samples. *P*‐value was calculated by Student's t‐test.


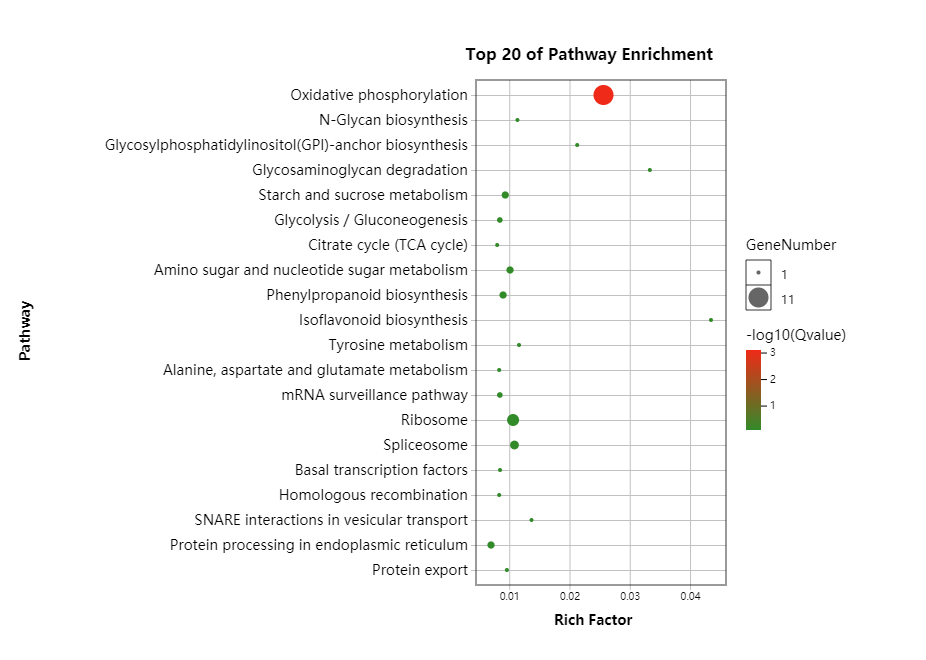


**Figure S10. Top 20 KEGG pathways of 318 nearest annotated genes from distal THSs.**


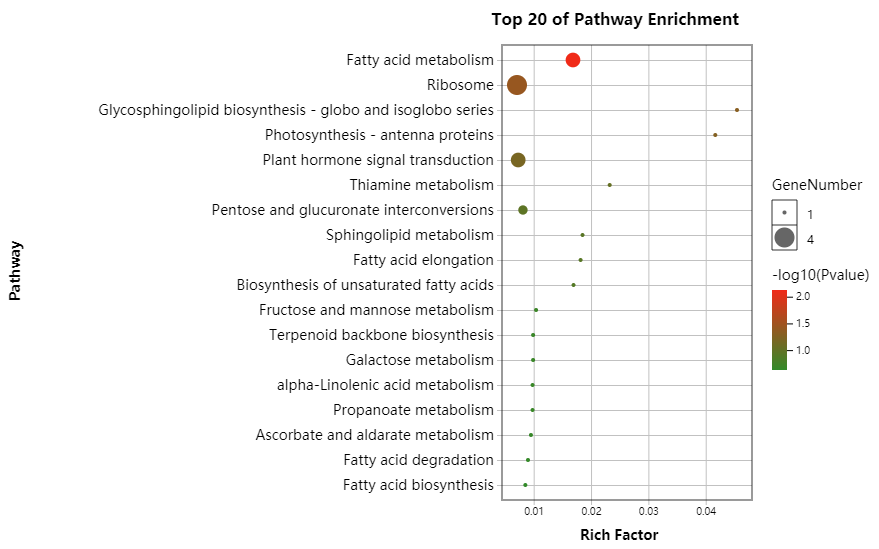


**Figure S11. Top 20 KEGG pathways of 66 nearest annotated genes from cold-induced THSs.**
